# Does gallic acid improve cardiac function by attenuation of oxidative stress and inflammation in an elastase-induced lung injury?

**DOI:** 10.22038/ijbms.2020.46427.10721

**Published:** 2020-09

**Authors:** Farzaneh Sohrabi, Mahin Dianat, Mohammad Badavi, Maryam Radan, Seyyed Ali Mard

**Affiliations:** 1 Department of Physiology, Physiology Research Center, Faculty of Medicine, Ahvaz Jundishapur University of Medical Sciences, Ahvaz, Iran

**Keywords:** Cardiovascular disease, Gallic acid, Hemodynamic parameters, Inflammation, Lung injury, Porcine, pancreatic, elastase, Rat

## Abstract

**Objective(s):**

Cardiovascular disease has an important role in mortality caused by lung injury. Emphysema is associated with impaired pulmonary gas exchange efficiency and airflow limitation associated with small airway inflammation. The aim was to evaluate the interactions between lung injury, inflammation, and cardiovascular disease. Since gallic acid has antioxidant and anti-inflammatory effects, we hypothesized that gallic acid protects the lung and the related heart dysfunction in elastase-induced lung injury.

**Materials and Methods:**

Forty-eight Sprague-Dawley male rats were randomly divided into six groups: Control, Porcine pancreatic elastase (PPE) , PPE+GA, and 3 groups for different doses of gallic acid (GA 7.5, GA 15, GA 30 mg/kg). PPE was injected intra-tracheally on days 1 and 10 of the test. In each group, electrocardiography, hemodynamic parameters, oxidative stress, and bronchoalveolar lavage fluid were examined.

**Results:**

PPE administration showed a decrease in HR and QRS voltage of electrocardiogram parameters, as well as in hemodynamic parameters (*P*<0.05, *P*<0.01, and *P*<0.001) and superoxide dismutase (SOD) (*P*<0.05). Tumor Necrosis Factor α (TNF-α) (*P*<0.001), interleukin 6 (IL-6) (*P*<0.001), interleukin 6 (MDA) (*P*<0.001), and the total number of white blood cells (*P*<0.001) showed an increase in PPE groups. Gallic acid preserved the values of hemodynamic properties, oxidative stress, inflammation, and electrocardiogram parameters in comparison to the PPE group.

**Conclusion:**

Briefly, this study showed the valuable effect of gallic acid in cardiac dysfunction related to elastase-induced lung injury. These findings suggested that gallic acid, as a natural antioxidant, has a potential therapeutic effect on preventing oxidative stress, inflammation, and subsequent cardiovascular disease.

## Introduction

Emphysema is one of the main disorders associated with chronic obstructive pulmonary disease (COPD). Emphysema is associated with the loss of the alveolar wall and its irreversible enlargement, restriction of airflow, depletion of lung elastin (1), and reduction of the gas exchanging surfaces of the lung (2). There is a prevailing opinion that COPD exacerbation is associated with chronic inflammation (3). Inflammation plays a fundamental role in the development of emphysema (4). Inflammation of the lung is primarily caused by the gradual infiltration of inflammatory cells, mainly macrophages, responsible for the degradation of alveolar elastin by secretion of elastinolytic proteases (5, 6). Besides the well-known effects of emphysema on the lungs, the systemic effects of emphysema have also been described (7). Inflammatory state in emphysema is not limited to the lungs, but it also affects systemic circulation and can affect non-pulmonary organs (8, 9). Acute and chronic lung inflammation is an unknown risk factor for cardiovascular disease (10). Chronic lung disease also has significant effects on right ventricular function and pulmonary artery pressure. The highest increase in mortality caused by COPD is related to the involvement of the heart and cardiovascular dysfunction (11). The cardiovascular system consists of systemic circulation, and the two right and left ventricular pumps. Two basic principles to remember for ventricular function are the fact that the left ventricle pumps blood via the aorta and the right ventricle blood to the pulmonary artery. When blood is not fully oxygenated due to emphysema, it can lead to stress on the heart tissue and cause symptoms of heart failure. On the other hand, excess fluid in the lungs caused by left heart failure can make the breathing process more difficult for individuals with COPD (12). Some studies have suggested a link between pulmonary disease and heart dysfunction (13). 

Since cardiac dysfunction plays a role in the overall complications of lung injury and chronic lung disease, it is essential to know how it plays a role in the treatment. There is a wide variety of plant-derived polyphenolic compounds. Their medicinal properties have received much attention in recent years. Gallic acid (3, 4, 5-trihydroxy benzoic acid) was highly regarded as a free radical scavenger and anti-oxidant (14). Gallic acid has anti-inflammatory (15), anti-oxidant (16), and anticancer effects (17).

In light of these findings, the aim of the presented study was to evaluate the hypothesis that gallic acid may affect oxidative stress and inflammatory processes to reduce inflammation and improve heart function in the elastase-induced-emphysema model. Furthermore, the electrocardiography (ECG) and hemodynamic parameters were assayed to evaluate the cardioprotective effects of gallic acid on isolated rat hearts in elastase-induced emphysema.

## Materials and Methods

Porcine pancreas elastase was purchased from Sigma-Aldrich (USA). Cytokine Elisa kits were purchased from ZellBio (Germany). Xylazine and ketamine were purchased from Alfasan (The Netherlands), Krebs salts from Merck (Germany), gallic acid, and heparin were bought from Sigma-Aldrich (USA). Male Sprague Dawley rats (weight range, 150 to 180 g) were classified into the 6 following groups (n=8): Control group, PPE group (intra-tracheal injection of 25 IU/kg body weight PPE on days 1 and 10 of the test) (18). Three groups treated with different doses of gallic acid (GA 7.5, GA 15, GA 30 mg/kg), were gavaged for 28 days (19), and the PPE group treated with GA intra-tracheal injection of PPE (25 IU/kg body weight) on days 1 and 10 of the test + Gallic acid (30 mg/kg/day), gavaged for 28 days. The experimental protocol was confirmed by the Animal Ethics Committee of Ahvaz Jundishapur University of Medical Sciences (APRC-9801).

***Experimental protocol***

The rats were anesthetized via IP injection of 50 mg/kg ketamine and 10 mg/kg xylazine (20) and then intra-tracheally injected with (25 IU/kg body weight) of porcine pancreatic elastase in saline (control) on days 1 and 10 of the test. The rats were killed 28 days after instillation. 

***Histopathological examination***

The left lobe of lungs was fixed in 10% formalin solution for 24 hr and then the tissues were stained with Masson’s trichrome to evaluate the deposition of collagen fibers (21).

***Extraction of alveolar bronchial lavage fluid***

In the end, anesthesia was induced by a mixture of ketamine and xylazine. After ensuring deep anesthesia, the chest was opened and the lung’s outer surface was rinsed with a small amount of saline and the trachea was cannulated. Then, 2 ml of phosphate-buffered saline (PBS) solution was administrated into the lung and immediately extracted with a suction syringe. This step was repeated for 5 series (22).

***Investigation of inflammatory factors***

For this purpose, the entire lavage fluid was centrifuged at 3000 rpm for 10 min at 4 ^°^C. Then the supernatant was removed and kept at -80 ^°^C until examination. The levels of IL-6 and TNF-α were measured using the ELISA (IBL, Germany) method according to the kit’s instructions (23).

***Measurement of the total number of white blood cells and differential counts in the alveolar bronchial lavage fluid***

For this purpose, a mixture of lung lavage solution was mixed with the same volume of the torque solution, and the total number of WBCs (white blood cells) was determined on a hemocytometer. The smears were prepared using cell pellet suspension. Then, dried and stained with the Wright-Giemsa solution. The total and differential inflammatory cell counts were determined with 400 magnifications (22). 

***Electrocardiogram (ECG) recording***

After anesthesia, lead II electrocardiogram was recorded by Bio-Amp and monitored by a Power Lab system (AD-Instruments, Australia) to determine heart rate, PR, QT, and RR interval, voltage of QRS, QRS interval and QTc (Bazett’s formula: QT interval/square root of the RR interval) (24). 

***Measuring the hemodynamic parameters of isolated hearts*** ***using Langendorff*** ***setup***

The animals were anesthetized with ketamine and xylazine. Heparin (1000 u/kg) was also used to prevent coagulation. Then the animals were connected to a ventilator (model: 7025, UGO BASILE), and the diaphragm was ruptured, a metal cannula (aortic cannula) inserted through the incision into the aorta, and the hearts were rapidly excised and mounted on a Langendorff setup with continuous retrograde perfusion using Krebs-Henseleit buffer consisting of glucose (11.1 mM), NaHCO_3_ (25 mM), KCl (4.75 mM), CaCl_2_ (1.75 mM), KH_2_PO_4_ (1.18 mM), MgSO_4_ (1.2 mM), and NaCl (118 mM) equilibrated by 5% CO_2_ and 95% O_2_ at pH of 7.4. The cardiac parameters such as HR (Heart rate), LVDP (Left ventricular developed pressure), LVEDP (Left ventricular end-diastolic pressure), LVSP (Left ventricular systolic pressure), max dp/dt (Maximum rate of rise (+dp/dt)) and min dp/dt (Minimum rate of fall (-dp/dt)), RPP (Rate pressure product) (HR × LVDP), were monitored continuously by a Power Lab system (AD Instruments) (25).

***Measurement of lipid peroxidation (MDA) and anti-oxidant enzyme (SOD) in heart tissue***

At the end of the experiment, 100 mg of heart tissue was homogenized in PBS. After centrifugation, the supernatant was separated to measure MDA and SOD. Then MDA and SOD were measured according to the ZellBio kit (26).

***Statistical analysis***

The results were described as mean ± SEM. All groups were statistically compared by ANOVA followed by Tukey’s multiple comparisons. *P*<0.05 was regarded as significantly different. 

## Results

***Confirmation of PPE-induced systemic inflammation***

**Figure 1:**
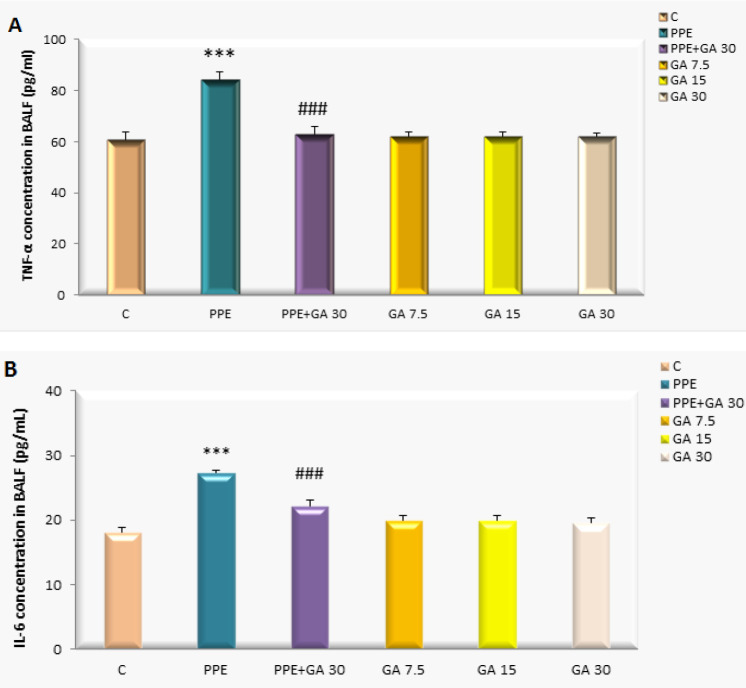
BALF contents of TNF-α and interleukine-6 in Control (C), PPE, PPE plus Gallic acid (GA 30), GA 7.5, GA 15, GA 30 mg/kg rats. ***P<0.001 vs the Control group; ###*P*<0.001 vs the PPE group. All groups were statistically compared by ANOVA followed by Tukey's multiple comparisons. Data are expressed as mean±SEM, n=8

BALF: bronchoalveolar lavage fluid; TNF-α: tumor necrosis factor α; PPE: porcine pancreatic elastase

The levels of TNF-α and IL-6 in BALF (bronchoalveolar lavage fluid) were examined in different groups to assess inflammatory changes (Figures 1A, 1B**)**. A significant increase in TNF-α and IL-6 was observed in the PPE group compared with the control (*P<0.001*). However, levels of TNF-α and IL-6 showed normal values in the PPE group using concomitant gallic acid (PPE+GA 30) (*P*<0.001).

***Inflammation cell count***

**Figure 2:**
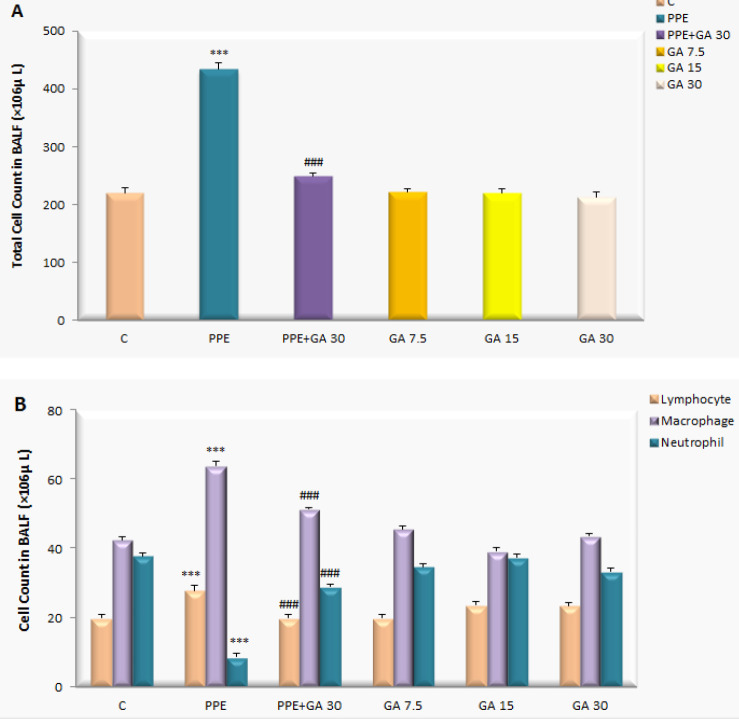
Total cell count and classification in bronchoalveolar lavage fluid (Control (C), PPE, PPE plus Gallic acid (GA 30), GA 7.5, GA 15, GA 30 mg/kg) rat. ***P<0.001 vs the Control group; ###*P*<0.001 vs the PPE group. Data are expressed as the mean±SEM, n=8. All groups were statistically compared by ANOVA followed by Tukey's multiple comparisons

To confirm lung inflammation and subsequent systemic inflammation, WBC (white blood cell) counts were performed (Figures 2A, 2B). In the PPE group, the total number of WBC and the numbers of macrophages and lymphocytes were significantly higher compared with the control group but neutrophils in the PPE group were lower compared with the controls (*P*<0.001). The BALF WBC including macrophages and lymphocytes in the PPE group co-treated with gallic acid (GA 30) significantly decreased (*P*<0.001) with respect to the PPE group while the neutrophils count in this group treated with gallic acid (GA 30) increased significantly (*P*<0.001).

***Changes in lung histology***

**Figure 3:**
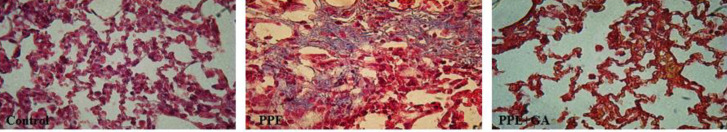
Lung histological changes in lung tissues of rat in Control, PPE, PPE+GA (30 mg/kg)

PPE: porcine pancreatic elastase; GA: gallic acid

As shown in Figure 3, in the lung tissue of emphysematous rats (PPE group) the deposition of collagen fibers increased compared with the control group, but gallic acid was able to decrease the deposition rate of collagen compared with the emphysematous group (PPE).

***Electrocardiographic parameters***

**Figure 4:**
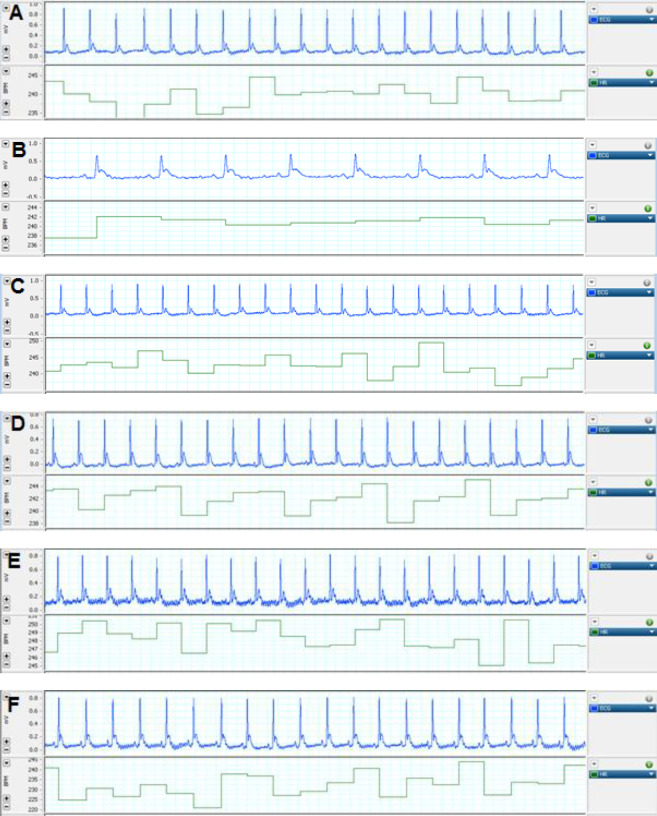
Electrocardiogram records from all rat groups. Control ( 4A), PPE (4B), PPE plus GA 30 (4C),GA 7.5 (4D), GA 15 (4E), and GA 30 mg/kg (4F). PPE: porcine pancreatic elastase; GA: gallic acid

To determine if PPE-induced emphysema has effects on the heart function, we compared cardiac electrocardiogram parameters between all groups. As shown in Table 1 and Figures 4A–4F, there was a decrease in HR (bpm) (*P*<0.001) and QRS complex (mv) (*P*<0.001) which is the negative inotropic indicator (myocardial contractility), and an increase in RR interval (S) (*P*<0.05), QRS Complex (S) (*P*<0.001), and QT interval (S) (*P*<0.05) in the PPE group. However, gallic acid could restore these changes significantly compared with the PPE group.

***Hemodynamic parameters***

**Figure 5:**
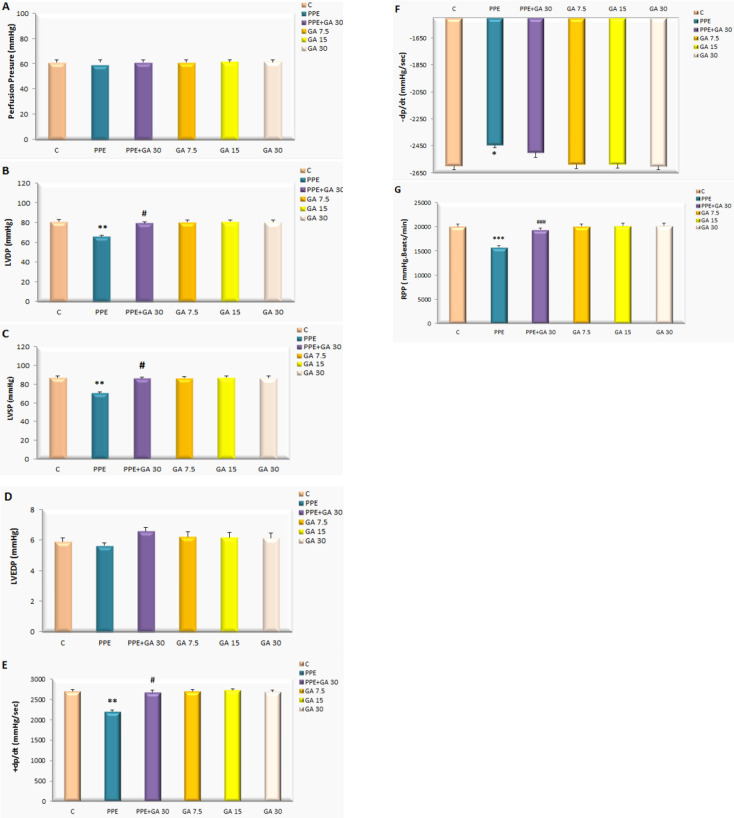
Hemodynamic parameters from isolated hearts in all rat groups. All groups were statistically compared by ANOVA followed by Tukey’s multiple comparisons. Data are expressed as mean±SEM (n=8). * *P*<0.05, ** *P*<0.01, *** *P*<0.001 vs the Control, # *P*<0.05, ### *P*<0.001 vs the PPE

PPE: porcine pancreatic elastase; GA: gallic acid; C: control

In consideration of the possible preventive effect of gallic acid on intrinsic cardiac damage induced by PPE, the hemodynamic parameters in isolated rat hearts were examined. Figures 5 A–5G show that there is a decrease in + dp/dt (mmHg) (*P*<0.01), - dp/dt (mmHg) (*P*<0.05), rate pressure product (RPP) (*P*<0.001), LVDP (mmHg) (*P*<0.01), and LVSP (mmHg) (*P*<0.01). HR (*P*<0.001) reduced in PPE compared with the control group. Gallic acid improved contractility of the heart and enhanced +dp/dt (mmHg) (*P*<0.05), LVSP (mmHg) (*P*<0.05), (RPP) (*P*<0.001), LVDP (mmHg) (*P*<0.05), and HR (*P*<0.01) in the emphysematous animals compared with the emphysematous animals that were not treated with gallic acid (Figure 5). In the PPE group, administration of gallic acid significantly restored these cardiac indexes. Other parameters showed no significant difference between different groups. 

***Levels of lipid peroxidation (MDA) and SOD in heart tissue***

**Figure 6:**
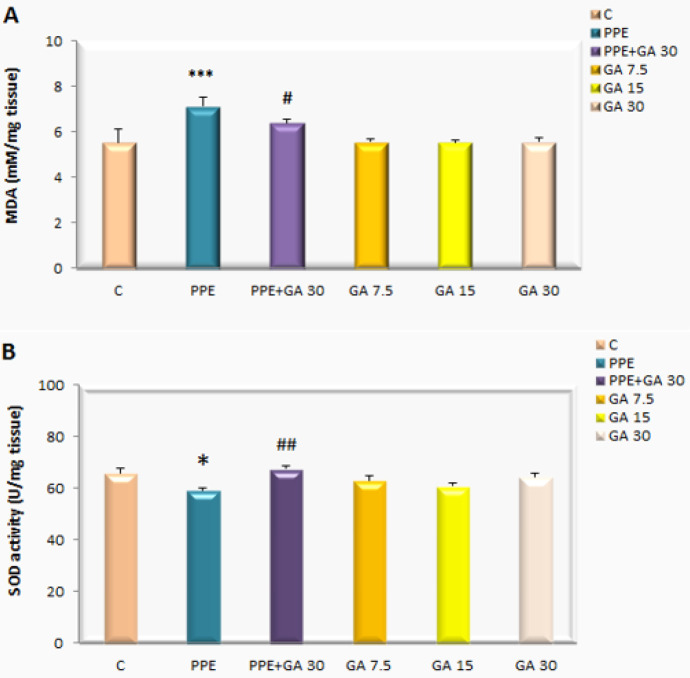
MDA levels and SOD in heart tissue rat in the groups of control, PPE, PPE+GA(30), GA (7.5, 15, and 30 mg/kg). *P<0.05 and ***P<0.001 vs control group, #*P*<0.05, ##*P*<0.01 vs PPE group. Data are expressed as mean±SEM (n=8)

MDA: malondialdehyde; SOD: superoxide dismutase; PPE: porcine pancreatic elastase; GA: gallic acid; C: control

The levels of MDA in the heart tissue increased significantly in the emphysematous group (PPE Group) compared with the control group (*P*<0.001) but gallic acid decreased levels of MDA in the emphysematous group (*P*<0.05). The activity of SOD in the heart tissue in the PPE Group decreased significantly compared with the control group (*P*<0.05). These levels were increased in the gallic acid-cotreatment group as compared with emphysematous rats (*P*<0.01) (Figure 6A, 6B).

## Discussion

This study provided clear evidence that gallic acid protects the heart against injury induced by the animal emphysematous model. However, elastase instillation is not used as a causing agent of human emphysema, unlike smoke exposure, it offers the advantages of being inexpensive, easy to obtain (27) and more causing widespread lung injury (28). In addition, administrations of elastase results not only in lung injury but also in extra-pulmonary injury (29), which is more similar to the real-life consequences of COPD patients (30). Therefore, elastase-induced emphysema models can be useful for studying treatment strategies (27). In the present study, emphysema was induced by intra-tracheal injection of porcine pancreatic elastase which leads to lung injuries and systemic disorders such as dysfunction in the cardiac electrocardiogram and hemodynamic parameters. This study investigates changes in cardiac function affected in the emphysema model induced by elastase.

Several studies have pointed to the link between COPD (of which emphysema is a part) and cardiovascular disease, noting that systemic inflammation may be shared in both COPD and cardiovascular disease (8, 31, 32). Half of the patients with COPD die from cardiovascular dysfunction (33). Chronic inflammation is essential for COPD and has a considerable effect on the pathogenesis of disease and progression (34). Increased inflammatory cells in BALF have been documented both in PPE-induced emphysema and in patients with this disease (35). Numerous studies in COPD patients demonstrated some changes in different inflammatory cells, including lymphocytes and neutrophils (36, 37). A study showed increased number of infiltrated inflammatory cells into the lung in an animal model of COPD (38). Another study demonstrated that the activity of cytochrome oxidase was increased in circulating lymphocytes in patients with COPD. This disorder could also be observed in circulating lymphocytes in patients with other chronic inflammatory diseases (39). In one study, macrophages and neutrophils subsequently accumulated in the alveolar spaces and consequently in progressive airspace enlargement in the first month after elastase induction (40). However, neutrophils are important in the pathology of COPD. In the present study, the number of neutrophils in the BALF was very low, which is consistent with the findings of another study (41). After elastase injection, neutrophilic inflammation generally happens and is resolved after the first week (42, 43). The time period (28 days) estimated in this research work confirms the low number of neutrophils in BALF. The number of leukocytes in BALF was higher than the control group, and macrophages showed the highest increase compared with lymphocytes. In accordance with the findings in this study, other studies using the elastase model have shown that most BALF cells are macrophages (41, 43). A study showed that alveolar macrophages have high elastinolytic activity in the lungs of smokers instead of neutrophils, suggesting that macrophages cause induction and development of emphysema in smokers and also in animals exposed to cigarette smoke (44). The role of macrophages in COPD was determined by Retamales *et al.* (2001) who suggested that macrophages become activated and release pro-inflammatory cytokines such as TNF-α and interleukins which enhance lung inflammation and promote disease progression (35). Also, in another study, it was demonstrated that monocytes produce more TNF-α in COPD patients compared with the control group (45). A study demonstrated that IL-6 was transmitted from the lungs to the systemic circulation, as shown by differences in arteriovenous IL-6 levels due to increased lung inflammation and simultaneous lung permeability (46). Also, a study showed that serum levels of IL-6 were significantly increased during the exacerbation of the disease, which may be more involved in cardiovascular morbidity and mortality in COPD patients (47). TNF-α is an inflammatory cytokine mainly produced by inflammatory reactions (48). Several studies have shown elevated levels of cytokines such as IL-6 and TNF-α in peripheral blood circulation in COPD patients (49-51). Another study showed that levels of TNF-α, IL-6, and IL-1β increased in sputum during emphysema in humans (52). Also, the present findings using an elastase-induced emphysema model indicate that the intra-tracheal administration of PPE can produce inflammation. Bronchoalveolar lavage fluid levels of TNF-α and IL-6 increased in emphysematous rats compared with the control group. We showed that gallic acid treatment reduced inflammatory cells such as macrophages and lymphocytes, and TNF-α and IL-6 levels, which probably affect the level of inflammatory cytokines. On the other hand, inflammation progression and oxidative stress are caused by the accumulation or overproduction of free radicals. Free radicals can cause lipid peroxidation (53). Malondialdehyde (MDA) is produced from peroxidation of lipids and is a marker of oxidative stress (54). The recent studies have shown that MDA is increased in people with COPD (55). Our study also showed a significant increase in MDA in emphysematous rats in comparison with the control group. Gallic acid significantly decreased the level of MDA. Decreased anti-oxidant levels or increased ROS levels induce oxidative stress. Anti-oxidant enzymes such as SOD have an important role in preventing oxidative stress (56). Our findings showed a significant decrease in the amount of SOD anti-oxidant enzyme in emphysematous rats but this enzyme is significantly increased by gallic acid.

On the other hand, Masson’s trichrome staining was done to evaluate the deposition of collagen and elastic fibers, and the emphysematous group showed a significant increase in collagen fibers in the lung parenchyma. These findings confirm previous studies (41, 57). A few studies are investigating the effect of phenolic compounds in COPD. The inhibitory effect of polyphenolic compounds on elastase activity was shown by Bras *et al.* (58). Another study demonstrated that resveratrol decreases cytokine production by macrophages in COPD patients (59).

Gallic acid is a potent natural anti-oxidant with high content of phenolic compounds that have strong anti-inflammatory and anti-oxidant effects (60), and it has also cardioprotective effects (61). Several studies documented the cardioprotective effects of gallic acid when rat hearts were exposed to some materials such as isoproterenol and aluminum oxide (62, 63). In this regard, Ramezani-Aliakbari *et al.* (2017) showed that gallic acid has a protective role in ameliorating left ventricular function in the alloxan-induced diabetes model (64). The results obtained by a study showed that gallic acid could prevent the inflammation process in the respiratory and cardiovascular system induced by ambient particulate matters (24). A group demonstrated that gallic acid improves the cardiac function and electrocardiographic irregularities induced by doxorubicin (65). Badavi *et al.* (2016) showed that gallic acid increased the QRS voltage and decreased the QTc interval in a model of liver cirrhosis following induction of bile duct ligation (66). Also, another study reported that gallic acid pretreatment ameliorated CaCl_2_-induced dysrhythmias (67). A study demonstrated that pre-treatment with gallic acid inhibited cardiotoxicity induced by isoproterenol (63). Also, another study reported that gallic acid increased the reduced hemodynamic parameters such as LVEDP, LVSP, LVDP, and RPP following I/R injury in hearts isolated from alloxan-induced diabetes mellitus (64). According to the results of this study, this is the first study that exhibited the significant adverse effects of elastase-induced emphysema on electrocardiogram and hemodynamic parameters. We observed that QRS complex voltage and heart rate reduced and following that QRS complex (s) and RR interval(s) showed a significant increase but the QT interval(s) showed a slight increase in emphysematous rats in comparison with the control group. Gallic acid significantly improved these parameters. Reduced QRS voltage causes the onset of factors such as myocardial injury (68). In the present study, we demonstrated a significant decrease in heart rate, LVDP, LVSP, +dp/dt, -dP/dt, and RPP in untreated rats of the PPE group, which is evidence of the development of severe myocardial injury. Gallic acid pretreatment preserves heart rate, LVDP, LVSP, +dp/dt, and RPP. Decreased LVDP and ±dp/dt (myocardial contraction and relaxation) is an index of reduction of contractile function (69). Also, LVSP can be considered as a marker for myocardial contraction (70). According to our findings, gallic acid increased myocardial contractility, improved hemodynamic parameters, and preserved ventricular function. This was shown by improvement in +dp/dt, resulting in increased cardiac output. Increased RPP is associated with enhancement of myocardial function and increased +dp/dt, which is considered an indicator of myocardial contractility (71).

PPE: porcine pancreatic elastase

**Table 1:** Electrocardiogram records from all rat groups

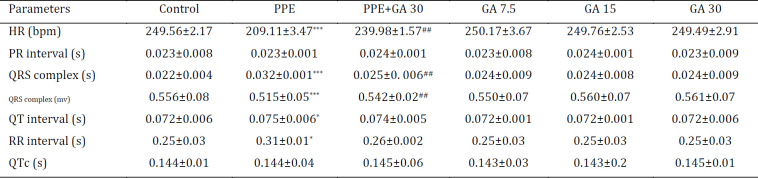

All groups were statistically compared by ANOVA followed by Tukey’s multiple comparisons. Data are expressed as mean±SEM (n=8).* *P*<0.05, *** *P*<0.001 vs the Control, ## *P*<0.01 vs PPE. PPE: porcine pancreatic elastase. ; GA: Gallic acid; HR: Heart rate

**Figure 7:**
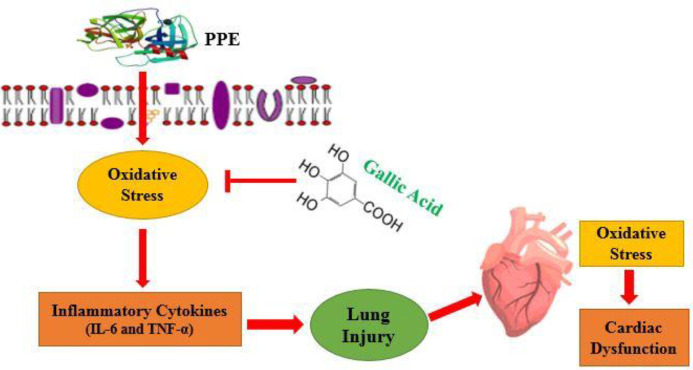
Schematic representation of the protective role of gallic acid against oxidative stress and inflammation pathway

## Conclusion

Briefly, in the present study, it was found that gallic acid has a potent therapeutic effect on cardiac dysfunction caused by elastase-induced lung injury such as emphysema, which may be associated with its anti-oxidant and anti-inflammatory properties (Figure 7).
